# Influence of Tableting on Enzymatic Activity of Papain along with Determination of Its Percolation Threshold with Microcrystalline Cellulose

**DOI:** 10.1155/2014/140891

**Published:** 2014-10-14

**Authors:** Manu Sharma, Vinay Sharma, Dipak K. Majumdar

**Affiliations:** ^1^Department of Pharmacy, Banasthali Vidyapith, Banasthali, Rajasthan 304022, India; ^2^Department of Bioscience and Biotechnology, Banasthali Vidyapith, Banasthali, Rajasthan 304022, India; ^3^Department of Pharmaceutics, Delhi Institute of Pharmaceutical Sciences and Research, Formerly College of Pharmacy, University of Delhi, Pushp Vihar, Sector III, New Delhi 110017, India

## Abstract

The binary mixture tablets of papain and microcrystalline cellulose (MCC), dicalcium phosphate dihydrate (DCP), carrageenan, tragacanth, and agar were prepared by direct compression. Carrageenan, tragacanth, and agar provided maximum protection to enzyme activity compared to MCC and DCP. However, stability studies indicated highest loss of enzyme activity with carrageenan, tragacanth, and agar. Therefore, compression behaviour of different binary mixtures of papain with MCC at different compaction pressures, that is, 40–280 MPa, was studied according to Heckel equation. The compressibility studies of binary mixtures indicated brittle behavior of papain. The application of percolation theory on the relationship between critical density as a function of enzyme activity and mixture composition revealed the presence of percolation threshold for binary mixture. Papain-MCC mixture composition showed significant percolation threshold at 18.48% (w/w) papain loading. Microcrystalline cellulose provided higher protection during stability study. However, higher concentrations of microcrystalline cellulose, probably as dominant particles, do not protect the enzyme with their plastic deformation. Below the percolation threshold, that is, 18.48% (w/w) papain amount in mixture with plastic excipient, activity loss increases strongly because of higher shearing forces during compaction due to system dominance of plastic particles. This mixture range should therefore be avoided to get robust formulation of papain.

## 1. Introduction

Among the new drug substances, the use of proteins and peptides as pharmaceuticals is steadily increasing, especially with evolution of recombinant DNA technique and advances in proteomics. Proteins possess greater biochemical and structural complexity compared to conventional drug based pharmaceuticals [1]. Thus, the formulation and delivery of proteins into stable well-characterised and efficacious drug products represent significant challenges to pharmaceutical scientist [2]. Tablets are suitable dosage form for application of these materials as they provide ease of administration, metering accuracy, robustness, good stability, and efficient production. However, simple compression of a bulk material, either powder or granulate, to a robust tablet is dependent on a great number of influences, mainly compression force, particle deformation, and formation of adhesive forces. Therefore, the physical and chemical properties of protein can be influenced by formulation and technological factors, for example, excipients, temperature, storage conditions, compression, or shear forces [3].

Pharmaceutical tablets generally comprise a number of components, which all contribute to the final properties of tablets. The properties of the tablets are usually predicted on behalf of the knowledge of properties of constituent substances. However, this is a challenging task due to the complexity and density of pharmaceutical blends. Therefore, an alternative approach would be to identify the dominant substance in terms of properties of interest and to predict the tablet properties from properties of two or more of these dominant constituents by the application of percolation theory [4]. Many tablet properties are related to the relative density of a tablet. The percolation theory relates changes in tablet properties to the existence of critical points in pharmaceutical formulations which can be related to the percolation threshold of a compact of the formulation [5, 6]. The knowledge of these critical points and corresponding percolation thresholds is of great importance to optimise the design of pharmaceutical dosage form. Furthermore, in order to prepare robust formulations, the neighbourhood of percolation thresholds should be avoided. Otherwise, a little change in the concentration of one component can cause a high variability in the properties of formulation.

Proteins in diet are essential for growth, repair, and for regulating the homeostasis of the body functions. But many people are intolerant to such foods including milk, cheese, yogurt, baked beans, bean soup, eggs, chicken, fish, and meat. So, the need for protein digesting supplement arises to overcome the deficiency manifestations [7]. Now a day's demand of protein digesting aids has increased; on the other hand, supply of pepsin (prepared from hog mucosa) has decreased. Thus, the plant source derived proteases like papain can be used as supplement as there will be no scarcity relative to its supply [8]. Papain is a food grade, highly active endolytic cysteine protease (EC 3.4.22.2) derived from* Carica papaya*. Its broad substrate specificity and the ability to hydrolyze small peptides as well as large proteins make papain an ideal enzymatic supplement [9]. Therefore, papain from* Carica papaya* was used as model enzyme in this work.

The approach of the present work is the compaction of enzyme (papain) to obtain more information about the tablet property, primarily the enzyme activity under pressure [10, 11]. Therefore, the main objective of the research work was detection of a percolation threshold to get more information of powder behaviour under compaction and to find out a mixture range in which the enzyme is protected by excipient.

## 2. Materials and Methods

### 2.1. Materials

Microcrystalline cellulose (MCC) (Avicel PH 102), dicalcium phosphate dihydrate (DCP), magnesium stearate, casein, tyrosine, and trichloroacetic acid (98.0%) were purchased from HiMedia Laboratories Pvt. Ltd., Mumbai, India. Potassium dihydrogen phosphate, sodium hydroxide (Qualigens Fine Chemicals, Mumbai, India) and papain (source* Carica papaya*), disodium ethylenediaminetetraacetate (99.5%), cysteine hydrochloride (99.0%), carrageenan, tragacanth, agar, and citric acid (98.0%) were purchased from S. D. Fine-Chem Ltd., Mumbai, India. All chemicals were used as received. Double-distilled water was used throughout the study.

### 2.2. Methods

#### 2.2.1. Enzyme Assay

The proteolytic activity of papain was estimated by modified casein digestion method of USP XXVII in the presence of cysteine hydrochloride. Appropriately diluted standard papain solution in phosphate-cysteine disodium ethylenediaminetetraacetate buffer was added to 5 mL of buffered 1% w/v casein substrate (pH 6.0 ± 0.1). After incubation at 37°C for 20 min in a shaking water bath, the reaction was stopped by the addition of 3 mL of 30% w/v trichloroacetic acid solution. The tubes were allowed to stand for 30–40 min at 40°C in water bath, to allow complete coagulation of the precipitated protein. Thereafter, the supernatant containing digested amino acids was filtered through Whatman filter paper number 42 by discarding first 3 mL of filterate. The absorbance of the filterate was measured at 280 nm using UV-VIS spectrophotometer (Lab-India UV 3000^+^, India) against the tyrosine standard plot of absorbance versus tyrosine concentration (*μ*g/mL). The papain activity was expressed in terms of casein digestion unit (CDU). A casein digestion unit (CDU) is the microgram of tyrosine liberated in 1 min by 1 mg enzyme under assay conditions.

#### 2.2.2. Influences on Enzyme (Papain) Activity

Enzymes are known to be sensitive to different stresses. Therefore, it was important to test if temperature or excipients could influence the activity of enzyme powder.

*Temperature. *The influence of different temperatures on the dry enzyme powder of papain was investigated. The dry powder was exposed to 30, 40, 50, 60, 70, and 80°C for 5 min, that is, the duration of compaction process. After this exposition, the enzyme activity was determined.

*Excipients. *The influence of various excipients, which have contact with the enzyme powder during powder compression, was investigated. Thus, dispersion of 50% enzyme with 50% MCC, DCP, carrageenan, agar, or tragacanth was prepared in phosphate buffer (pH 6.0) and the enzyme activity was detected and compared with reference.

#### 2.2.3. Preparation of Tablets of Enzyme with Different Excipients

Binary mixtures of enzyme (papain) with MCC, DCP, carrageenan, agar, and tragacanth were prepared in 1 : 1 ratio, respectively. The powders were mixed manually for 5 min. Samples comprising 200 mg of binary mixture powder were manually filled into a die of 8 mm in diameter (Cadmach Machinery Private Ltd., Ahmedabad) at 80 MPa compression pressure and the powders were compressed. Different batches of tablets prepared with various excipients were coded as given in Table 1. For each system 20 tablets were compressed. From the compression process only out-of-die data were generated.

#### 2.2.4. Stability Study of Tablets

Different batches of tablets were packed in amber coloured glass bottles and subjected to stability testing according to the International Conference on Harmonization guidelines for zones III and IV. The packed containers were kept for accelerated (40 ± 2°C/75 ± 5% relative humidity) and long-term (30 ± 2°C/65 ± 5% relative humidity) stability for 6 months and 12 months, respectively. Samples kept under accelerated storage conditions were withdrawn at 0, 1.5, 3, and 6 months and papain activity was estimated. Similarly, samples stored at 30 ± 2°C/65 ± 5% were withdrawn at 0, 3, 6, 9, and 12 months and analysed for papain activity. Visual inspection of samples for discoloration of tablet content was also done after completion of stability study.

#### 2.2.5. Compaction Behaviour of Enzymes with MCC

*Preparation of Compact with MCC. *Compacts of papain, MCC, DCP, and binary mixtures of MCC and papain were prepared, respectively. Binary mixtures of MCC and enzyme were prepared with different fractions of constituent components. These binary mixtures consisted of different amounts of papain, that is, 1, 20, 40, 60, and 80% (w/w). The powders were mixed manually for 5 min. Samples comprising 200 mg of binary mixture powder were manually filled into a die of 8 mm in diameter (Cadmach Machinery Private Ltd., Ahmedabad) and the powders were compressed and decompressed without holding between the compression and decompression stages. Tablets with different densities were prepared by varying the total compression pressure ranging from 40 MPa to 280 MPa. For each system and compaction pressure, 20 tablets were compressed.From the compression process only out-of-die data was generated.

*Determination of Relative Density and Porosity of Compact. *The tablet weight was measured using an electronic balance (Shimadzu AUY 220, Japan). The diameter and the thickness were measured with vernier calliper after a storage time of 24 h at 45% relative humidity in a desiccator. From these measurements, the apparent volume of the tablets was determined. The apparent density, *ρ*_*a*_, was then determined by

(1) $$\begin{array}{c} \rho_{a}=\frac{\text{Tablet}\;\text{weight}}{\text{Apparent}\;\text{volume}\;\left(V_{\text{tot}}\right)}. \end{array}$$

The true density (*ρ*_*t*_) of the powder was measured, using a helium gas displacement pycnometer (Type AccuPyc 1330, Micromeritics, Bedfordshire, UK).

The relative density (*ρ*_*r*_) was calculated by dividing the tablet apparent density by the true density of the binary mixture of powders used:

(2) $$\begin{array}{c} \rho_{r}=\frac{\rho_{a}}{\rho_{t}}=\frac{V_{t}}{V_{\text{tot}}}.\; \end{array}$$

*V*_*t*_ characterises the true volume of the solid particles and therefore (2) shows that the relative density is essentially a solid fraction.

The relative porosity (*ε*) of the compact is then calculated as

(3) $$\begin{array}{c} \varepsilon =\frac{\left(V_{\text{tot}}-V_{t}\right)}{V_{\text{tot}}}=1-\frac{V_{t}}{V_{\text{tot}}}=1-\rho_{r}. \end{array}$$

#### 2.2.6. Heckel Analysis

The compaction characteristics of the powder were studied by plotting −ln⁡⁡(porosity) versus compaction pressure according to Heckel equation [12]:

(4) $$\begin{array}{c} \ln\left(\frac{1}{1-\rho_{r}}\right)=KP+A, \end{array}$$

where *ρ*_*r*_ is the relative density of the compact, *P* is the applied pressure, and *K* (the slope of the linear portion) is the reciprocal of the yield pressure, *P*_*y*_, of the material. The yield pressure is inversely related to the ability of the material to deform plastically under pressure and *A* is a function of the original compact volume.

#### 2.2.7. Determination of Percolation Threshold

According to the percolation theory, critical normalised density (a function of tablet property) of various binary systems was determined by plotting enzyme activity as a function of normalised density (apparent density/poured density) of compact under different compression pressure [5]. The critical normalised density of various systems was determined by dividing the data in the curve into two straight lines using the following equation:

(5) $$\begin{array}{c} y=A\left(m_{1}x+b_{1}\right)+B\left(m_{2}x+b_{2}\right), \end{array}$$

where *x* is the relative density of compact, *y* is compaction pressure, *m* is the slope, *b* is intercept, and *A* and *B* are constants. The data points around the critical value shown as a bend in the curve were attributed alternatively to the first section or to the second section. The final attribution was made considering the correlation coefficient *R* ^2^ for the overall fit.

The critical normalised density (a function of tablet property) changes in the vicinity of percolation threshold of material in binary mixture [13]. Equation (5) was used for plotting critical normalised density of various systems against the percentage concentration of papain in binary mixture to determine percolation threshold of different binary mixtures of papain and microcrystalline cellulose.

## 3. Results and Discussion

### 3.1. Influences on Enzyme Activity

The influence of temperature and different excipients on the enzyme (papain) activity was tested. These investigations provided the information concerning the stability of the enzyme powder useful for characterisation of the enzyme powder.

#### 3.1.1. Temperature

**Figure 1:**
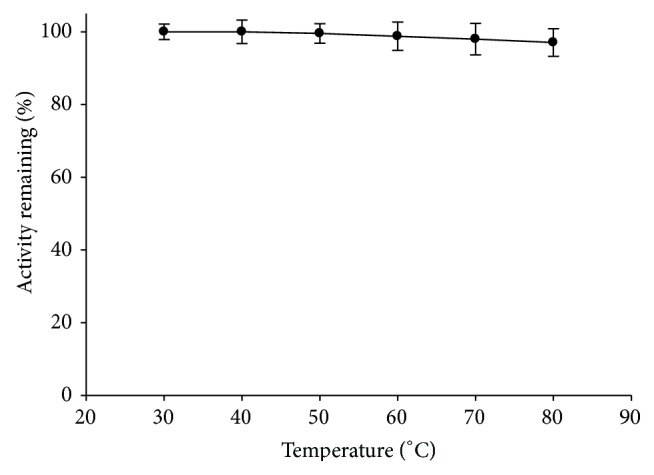
Effect of exposure of dry enzyme powder to different temperatures (30–80°C) for 5 min on the activity of enzyme.

The exposure of dry enzyme powder to different temperatures for the time of a compaction process had not showed any significant change in the activity from 40 to 80°C (Figure 1). The problems are not expected to arise from temperature development in die for tablet compaction of dry enzyme powder. Temperature rise over 80°C during compression is not probable [14, 15]. Hence, possible activity loss during compression may not derive from warming in die.

#### 3.1.2. Excipients

The behaviour of the activity of papain in the presence of various excipients was investigated and compared to the activity of papain powder as reference. The difference between the reference and the solutions of papain powder and excipient, that is, MCC, DCP, carrageenan, agar, or tragacanth, was not significant as the activity of enzyme which remained almost the same. Hence, influence of these excipients on the activity of papain is not evident.

### 3.2. Enzyme Activity of Tablets

Compacts of papain along with various excipients, that is, MCC, DCP, carrageenan, agar, and tragacanth, showed decrease in enzyme activity, respectively, when compared with the reference standards (Table 2). The greater loss in activity was observed with DCP and MCC compared to carrageenan, agar, and tragacanth.

### 3.3. Stability Studies of Tablets

Tables 3 and 4 present the results of accelerated and long-term stability studies of compacts and bulk papain formulations. The tablet formulations PM and PD showed around 87% papain content on storage under accelerated conditions (i.e., 40°C/75% RH) for 6 months, while PC, PA, PT, and PP tablet formulations showed around 83% and 75% papain content, respectively (Table 3). PM and PD, however, showed around 88% drug content when stored at 30°C/65% RH for 12 months against 74.87% drug content for PP formulation. PC, PA, and PT formulations showed around 84% drug content when stored under similar conditions for 12 months. The results suggest improved stability of the enzyme on compaction with excipients. On the basis of first-order degradation rate constants, the calculated *t*_90_ of PM, PD, PC, PA, and PT at 30°C/65% RH would be 327, 304, 224, 217, and 214 days, respectively (Table 4).

The *K*_calc_/*t*_90_ values suggest that formulations will not provide 1 year shelf life (*t*_90_) of the product and would need larger amount of overages resulting in higher initial drug concentration. The colour of papain compacts along with carrageenan, agar, and tragacanth changed from pale buff to dark brown, whereas papain compact formulations with MCC and DCP changed from pale buff to light brown. Thus, the stability of papain compacts along with excipients like carrageenan, agar, and tragacanth was significantly lesser than compacts with MCC and DCP. The loss of enzyme activity with carrageenan, agar, and tragacanth was more because of their greater moisture adsorption capacity. Thus, stability of compacts can be enhanced if protection is given against moisture to the compacts. However, greater amount of decrease in activity was observed during compaction of enzyme (papain) with MCC compared to carrageenan, agar, and tragacanth. Therefore, it becomes imperative to study the compression behaviour of enzymes, that is, papain along with MCC.

### 3.4. Compression Behaviour

**Figure 2:**
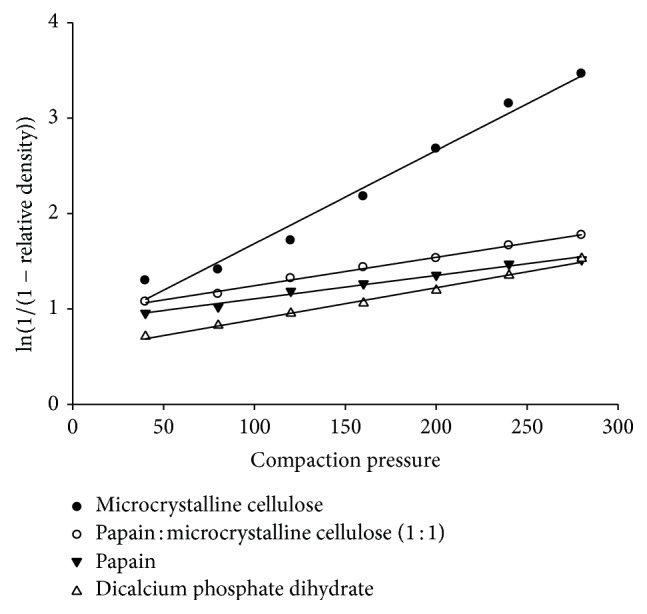
Heckel plots of comparison of papain, dicalcium phosphate dihydrate, microcrystalline cellulose, and papain-microcrystalline cellulose binary mixture (1 : 1).

According to Heckel analysis, on plotting −ln⁡⁡(porosity) against compaction pressure, it was observed that papain and DCP behave very similarly contrary to MCC (Figure 2). Slopes of curves (*K* values) of papain and DCP were 0.0057 MPa^−1^ (*R* ^2^ = 0.986) and 0.0079 MPa^−1^ (*R* ^2^ = 0.994), respectively, whereas the *K* value of MCC was 0.0226 MPa^−1^ (*R* ^2^ = 0.980). *K* value for MCC confirms the fact that plastic substances have higher *K* values than brittle substances [12, 16]. As DCP is known to have brittle properties, papain powder can be classified as brittle substance. However, the comparison of MCC with binary mixture of papain-MCC in 1 : 1 ratio showed that there is no linear relationship between the Heckel plot of excipient alone and along with enzyme. A slight dominance of papain in the Heckel plot was seen (Figure 2). The *K* values confirmed the statement, that is, *K* = 0.0071 MPa^−1^, 0.0226 MPa^−1^, and 0.0057 MPa^−1^, for binary mixture of papain-MCC (1 : 1), MCC, and papain, respectively.

**Figure 3:**
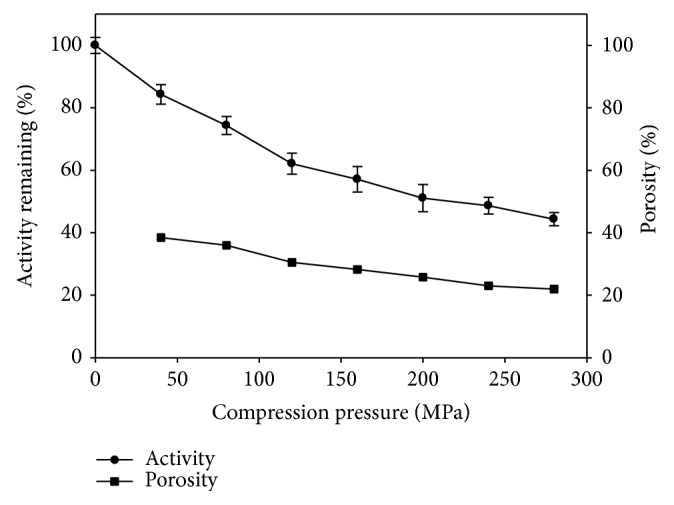
Relationship between % papain activity remaining and porosity of tablet with increase of compression pressure.

The behaviour of the enzyme under compression was further characterized by determining the activity loss at different compression pressure (Figure 3). The enzyme activity decreased steadily up to a compaction pressure of 160 MPa, whereas, at higher compaction pressures, the curve flattened and the degree of the activity loss decreased. Comparison with the porosity of the tablets showed that the curve also flattened slightly after the compression pressure of 160 MPa. The correlation coefficient *r* of activity of papain with the porosity of their respective tablets showed a value of 0.991, suggesting a positive correlation. This correlation can be explained with the reduction of interparticle space, which is big in the stage of particle movement and rearrangement and diminishes in the stage of particle deformation and therefore by a destruction of the native state of the enzyme under compression. This destruction is probably linear in the stage of particle movement and rearrangement. In the stage of deformation, that is, the region of the flattening slope, shearing forces will probably decrease as a consequence of the reduced particle movement.

### 3.5. Determination of Critical Density

**Figure 4:**
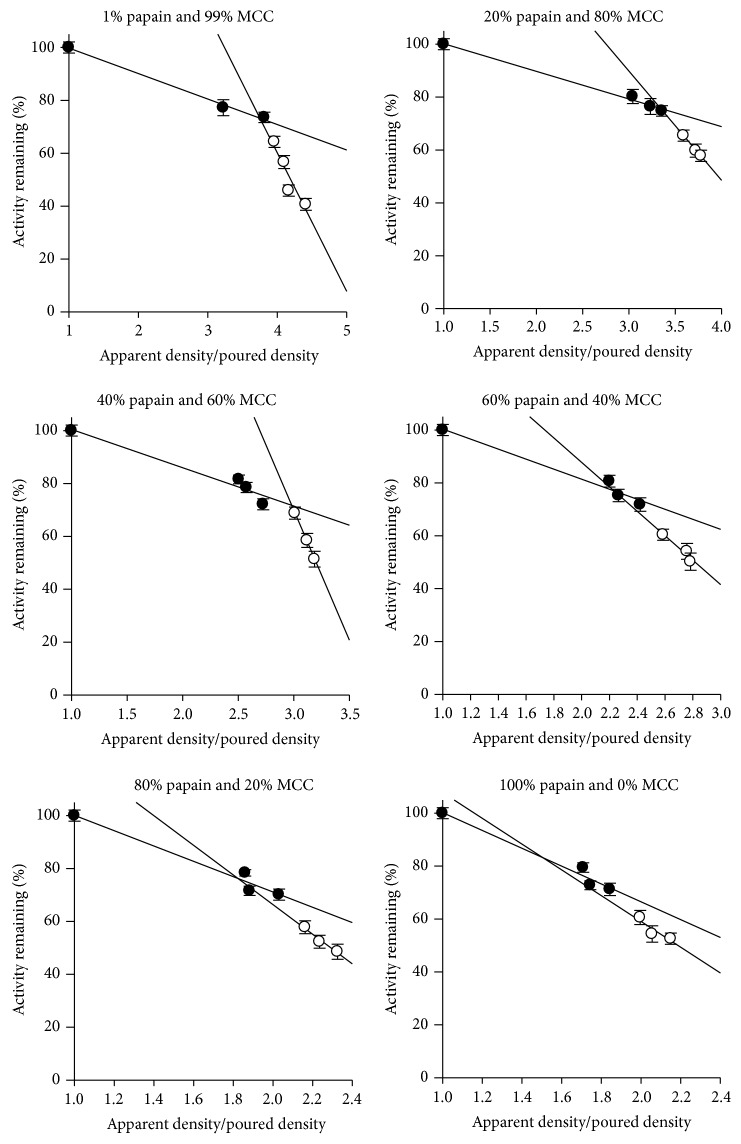
Relationship between papain activity remaining (%) in tablet with density on compaction of different binary mixtures of papain with microcrystalline cellulose (MCC).

Enzyme activity was analyzed as a function of the apparent density of compact. The apparent density of compact is a result of compaction pressure applied. Before compression, 100% of enzyme activity was observed for the material. The apparent density of compact was normalized with the poured density of material before compression. Thus, for the normalised relative density *ρ*_*n*_ = 1, 100% enzyme activity, and for the higher values for *ρ*_*n*_, the respective value of enzyme activity was measured. The relationship between activity and density of the compacts analysed with different composition of binary mixture of excipient and enzyme (papain) is shown in Figure 4. Equation (5) was used to approximate the curves so obtained with different binary mixtures with two linear sections and each section was linearised by two regression lines. The intersection of two regression lines was determined as critical normalised density. Thus, for each mixture, a critical normalised density was obtained, where the enzyme activity loss becomes more important. At higher compaction pressures, the degree of activity loss is more [17]. Figure 4 also shows that the activity loss at very high pressures depends only on the final apparent density of the compact (out-of-die).

**Figure 5:**
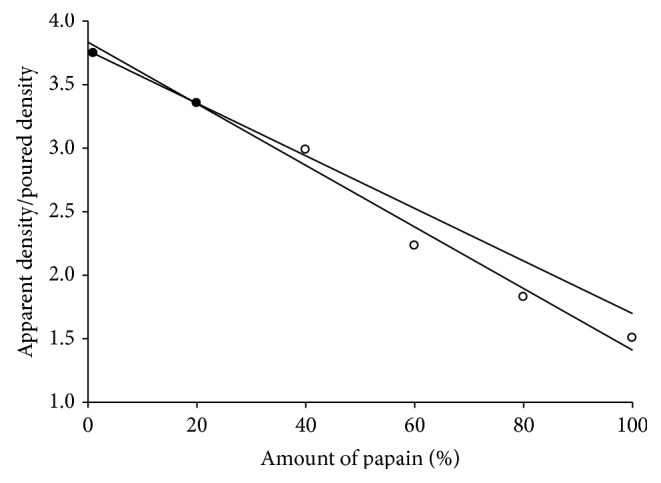
Relationship between critical densities of each binary mixture of papain with microcrystalline cellulose and the amount of papain (%).

**Figure 6:**
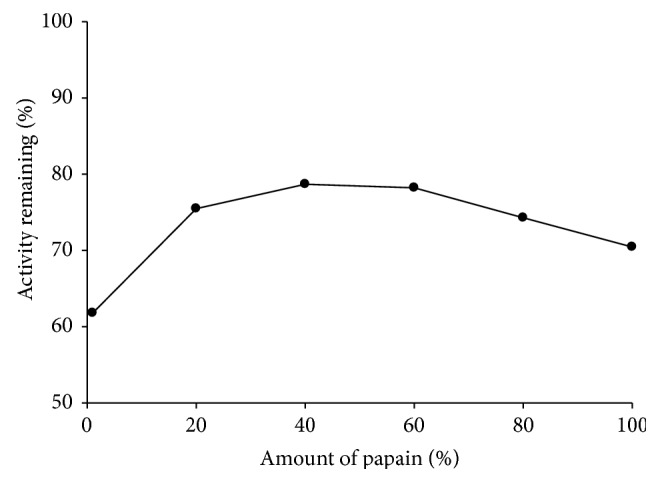
Relationship between percentage enzyme activity remaining and the amount of enzyme (%).

The detected critical normalized densities of various binary papain-MCC powder mixtures were plotted as a function of the amount of papain (Figure 5). The plot was divided into two sections with a squared correlation coefficient *R* ^2^ of 0.999 for section 1 and 0.959 for section 2 (Figure 5), respectively. The point of intersection, that is, 18.48% (w/w) (Figure 5), indicates the critical concentration of papain for binary mixtures with MCC. This critical papain concentration can be interpreted as the percolation threshold of these binary mixture systems. It defines the point where the system dominance of MCC is replaced by the dominance of papain. Above the percolation threshold, the brittle substance (papain powder) builds a lattice. The rigidity of the lattice and the filling of pore spaces with MCC particles prevent the fracturing of the brittle particles and therefore reduce the shearing forces in the compact. This seems to be favourable and reduces the loss of enzyme activity (Figure 6).

Below the percolation threshold, the enzyme is completely embedded in the MCC, that is, in the plastic material. As the compaction pressure applied, tablet can be compacted to higher density because of plastic flow of material. Thus, tablet porosity is reduced along with increased activity loss as rigid lattice of the enzyme powder is destroyed. In addition, the shearing forces are higher with a dominance of MCC because particle movement is bigger as compared to lattice dominated by the brittle substance. This mixture range should therefore be avoided to get robust formulations because a sudden change in the behaviour of the system occurs at this mixture range. Thus, the plastic powder (MCC) does not work as the protecting substance during compression.

## 4. Conclusions

The activity of investigated model enzyme papain was negatively influenced by the application of compaction pressure, whereas the extent of activity loss was dependent on the compression character of compacted substances. Compression properties of papain studied with excipients showed that enzyme powder compressed alone had greater loss in enzyme activity compared to combinations with excipients like carrageenan, tragacanth, and agar. On the contrary, MCC and DCP did not show protection to enzyme activity similar to carrageenan, agar, and tragacanth. However, MCC provided higher protection during stability study. On the other hand, MCC in higher concentration, probably as the dominant particles, do not protect the enzyme with their plastic deformation but they do possibly crush the particles of the enzyme powder and cause high shearing forces on the enzyme. The percolation threshold, that is, the change in the system dominance, was found to be at a mixture ratio of 18.48 (w/w) of papain enzyme powder. Below the percolation threshold, that is, <20% (w/w) for papain powder, there was steep decrease in enzyme activity. This mixture range should therefore be avoided to get robust formulations because a sudden change in the behaviour of the system occurs at this range and the expected protecting effect of the plastic excipient on the brittle papain enzyme powder was therefore not found.

## Figures and Tables

**Table 1:** Different batches of tablets prepared with MCC, DCP, carrageenan, agar, and tragacanth.

Excipients	Excipient : enzyme ratio	Formulation code for papain tablets
Nil	—	PP
MCC	1 : 1	PM
DCP	1 : 1	PD
Carrageenan	1 : 1	PC
Agar	1 : 1	PA
Tragacanth	1 : 1	PT

^*^PP represents tablets of papain powder.

**Table 2:** Effect of compaction of enzymes (papain) with different excipients on activity of enzymes.

Formulation	% activity remaining
PP	85.11 ± 3.82
PM	83.56 ± 4.01
PD	82.45 ± 3.67
PC	91.67 ± 4.32
PA	88.67 ± 3.81
PT	90.34 ± 2.76

^*^Values are mean ± SE (*n* = 3).

**Table 3:** Stability of papain powder tablet alone and in combination with different excipients under accelerated storage conditions (40 ± 2°C/75 ± 5% RH).

Formulation	% activity remaining				*K* (days^−1^)
	0 M	1.5 M	3 M	6 M	
PP	100.00 ± 3.12	94.07 ± 2.75	83.56 ± 3.18	75.84 ± 2.87	1.54 × 10^−3^
PM	100.00 ± 2.61	97.54 ± 2.13	94.53 ± 1.66	88.69 ± 2.19	6.67 × 10^−4^
PD	100.00 ± 3.48	96.21 ± 2.67	93.50 ± 3.61	87.14 ± 4.62	7.65 × 10^−4^
PC	100.00 ± 2.24	96.18 ± 4.51	92.45 ± 3.81	83.62 ± 3.26	9.94 × 10^−4^
PA	100.00 ± 3.28	94.41 ± 1.94	90.32 ± 4.45	82.96 ± 5.14	1.04 × 10^−3^
PT	100.00 ± 3.09	95.04 ± 1.93	91.74 ± 4.19	83.06 ± 4.11	1.03 × 10^−3^

^*^Values are mean ± SE (*n* = 3); M: months; *K*_cal_: calculated first-order degradation rate constant.

**Table 4:** Stability of papain tablet alone and along with different excipients under room temperature storage (30 ± 2°C/65 ± 5% RH).

Formulation	% activity remaining					*K* (days^−1^)	*t*_90_ (days)
	0 M	3 M	6 M	9 M	12 M		
PP	100.00 ± 3.16	95.56 ± 4.61	88.97 ± 3.48	81.48 ± 4.62	74.87 ± 4.64	7.93 × 10^−4^	131.14
PM	100.00 ± 2.61	96.71 ± 2.61	94.08 ± 2.04	91.80 ± 2.22	89.07 ± 2.44	3.17 × 10^−4^	327.89
PD	100.00 ± 3.13	97.16 ± 2.61	94.78 ± 2.49	92.08 ± 3.82	88.27 ± 3.27	3.42 × 10^−4^	304.18
PC	100.00 ± 3.06	96.12 ± 3.19	92.04 ± 3.78	89.80 ± 2.93	84.47 ± 1.77	4.62 × 10^−4^	224.88
PA	100.00 ± 2.13	97.01 ± 2.18	94.08 ± 4.04	88.80 ± 4.20	84.01 ± 3.56	4.77 × 10^−4^	217.83
PT	100.00 ± 4.01	96.01 ± 2.69	91.81 ± 2.74	87.23 ± 3.43	83.76 ± 1.77	4.86 × 10^−4^	214.16

^*^Values are mean ± SE (*n* = 3); M: months; *K*_cal_: calculated first-order degradation rate constant; *t*_90_: time to reach 90% of initial drug concentration.

## References

[1] Semalty A., Semalty M., Singh R., Saraf S. (2007). Properties and formulation of oral drug delivery systems of protein and peptides. *Indian Journal of Pharmaceutical Sciences*.

[2] Frokjaer S., Hovgaard L., Weert M. V. D., Mahato R. I. (2005). Formulation, stability and characterization of protein and peptide drugs. *Biomaterials for Delivery and Targeting of Proteins and Nucleic Acids*.

[3] Manning M. C., Patel K., Borchardt R. T. (1989). Stability of protein pharmaceuticals. *Pharmaceutical Research*.

[4] Leuenberger H., Rohera B. D., Haas C. (1987). Percolation theory—a novel approach to solid dosage form design. *International Journal of Pharmaceutics*.

[5] Blattner D., Kolb M., Leuenberger H. (1990). Percolation theory and compactibility of binary powder systems. *Pharmaceutical Research*.

[6] Leuenberger H. (1999). The application of percolation theory in powder technology. *Advanced Powder Technology*.

[7] Dobrilla G. (1989). Management of chronic pancreatitis: focus on enzyme replacement therapy. *International Journal of Pancreatology*.

[8] http://www.nutriteck.com/papain.html.

[9] Arnon R. (1970). Papain. *Methods in Enzymology*.

[10] Zarrintan M. H., Teng C. D., Groves M. J. (1990). The effect of compactional pressure on a wheat germ lipase preparation. *Pharmaceutical Research*.

[11] Wurster D. E., Ternik R. L. (1995). Pressure-induced activity loss in solid state catalase. *Journal of Pharmaceutical Sciences*.

[12] Heckel R. W. (1961). An analysis of powder compaction phenomena. *Transactions of the Metallurgical Society of AIME*.

[13] Holman L. E., Leuenberger H. (1990). The effect of varying the composition of binary powder mixtures and compacts on their properties: a percolation phenomenon. *Powder Technology*.

[14] Travers D. N., Merriman M. P. (1970). Temperature changes occurring during the compression and recompression of solids. *Journal of Pharmacy and Pharmacology*.

[15] Wurster D. E., Creekmore J. R. (1986). Measurement of the thermal energy evolved upon tablet compression. *Drug Development and Industrial Pharmacy*.

[16] Heckel R. W. (1961). Density—pressure relationship in powder compaction. *Transactions of the Metallurgical Society of AIME*.

[17] Morii M., Sano A., Takeguchi N., Horikoshi I. (1973). Studies on inactivation of alkaline protease by tabletting. *Yakugaku Zasshi*.

